# Procedure Planning: Anatomical Determinants of Strategy

**DOI:** 10.2174/1573403X10666140331142805

**Published:** 2014-05

**Authors:** Colm Hanratty, Simon Walsh

**Affiliations:** 1 Department of Cardiology, Belfast Health and Social Care Trust, Lisburn Road, Belfast. N Ireland. BT9 7AB, Ireland

**Keywords:** Antegrade dissection re-entry, chronic total occlusion, hybrid algorithm, percutaneous coronary intervention.

## Abstract

In contemporary practice there are three main methods that can be employed when attempting to open a chronic total occlusion (CTO) of a coronary artery; antegrade or retrograde wire escalation, antegrade dissection re-entry and retrograde dissection re-entry. This editorial will attempt to clarify the anatomical features that can be identified to help when deciding which of these strategies to employ initially and help understand the reasons for this decision.

## INTRODUCTION

In contemporary practice there are three main methods that can be employed when attempting to open a chronic total occlusion (CTO) of a coronary artery; wire escalation - either antegradely or retrogradely, antegrade dissection re-entry (ADR) and retrograde dissection re-entry (RDR). This editorial will attempt to clarify the anatomical features that can be identified to help when deciding which of these strategies to employ initially. 

Previously coronary angiographic anatomical features were used to determine whether it was feasible to open a CTO. Nowadays the anatomical features of a CTO are mainly used to plan the procedure and guide the initial strategy. Whether to open a CTO or not should be driven solely by clinical indications such as the presence of angina, significant demonstrable ischaemic burden and viability in the territory supplied by the CTO. Indications and justification for opening a CTO are covered in a companion article in this journal.

Previously described predictors of feasibility – a blunt stump or the presence of side branch at the proximal cap, significant calcification anywhere within the occlusion, vessel bending or tortuosity within the occlusion and the occlusion length (> than or < than 20 mm) [1] still predict escalating technical challenges in recannalizing the vessel. However, with the exception of the occlusion length these features do not affect the initial procedural strategy. Therefore, these variables may predict anticipated difficulty in successfully opening the CTO, but should not preclude an attempt. In particular they can identify sub-groups (easy or 0 / 1 J-CTO score) where an experienced CTO operator would expect to have crossed the lesion within a relatively short time (under 30 mins >90% of the time). They may also be used to identify patients where it may be reasonable to refer to high volume CTO experts (high J-CTO scores) to increase likelihood of initial success and reduced need for repeat procedures. 

**Fig. (1):**
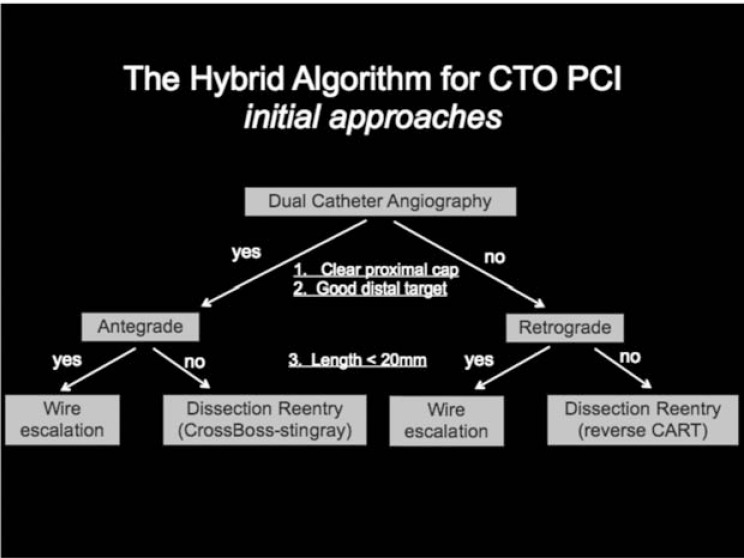
The Hybrid Algorithm.

The Hybrid CTO strategy algorithm is the current template for contemporary CTO practice [2] (Fig. **1**). This algorithm relies on 4 pieces of anatomical information which can be obtained from a good quality diagnostic coronary angiogram. These are: the proximal cap, the length of the occlusion (greater or less than 20 mm long), the presence of interventional collaterals, the distal cap and distal landing zone. The importance of these features will be discussed throughout this review but initial angiography must include the information relating to these points. For example it may be necessary to alter the camera angles to clearly visualise the proximal cap and its relationship to branches or collaterals. Long acquisition runs are required to demonstrate collateral filling of the target vessel, to identify the dominant collateral eg septal or epicardial and to determine whether these are suitable for crossing with guidewires and microcatheters (ie are “interventional collaterals”). This is best achieved on lower magnification, without panning of the table so the direction of collateral filling can be identified. In addition, the pattern of filling of the target vessel should also be examined carefully where there are several collaterals present. This can provide information about collateral dominance and also help operators to understand if there is continuity between distal branches in the target vessel. Therefore, the angiographic angle for acquisition is important as is the correct collimation. 

There are 4 angiographic anatomical features that influence the initial strategy when using the Hybrid Algorithm.

**Fig. (2):**
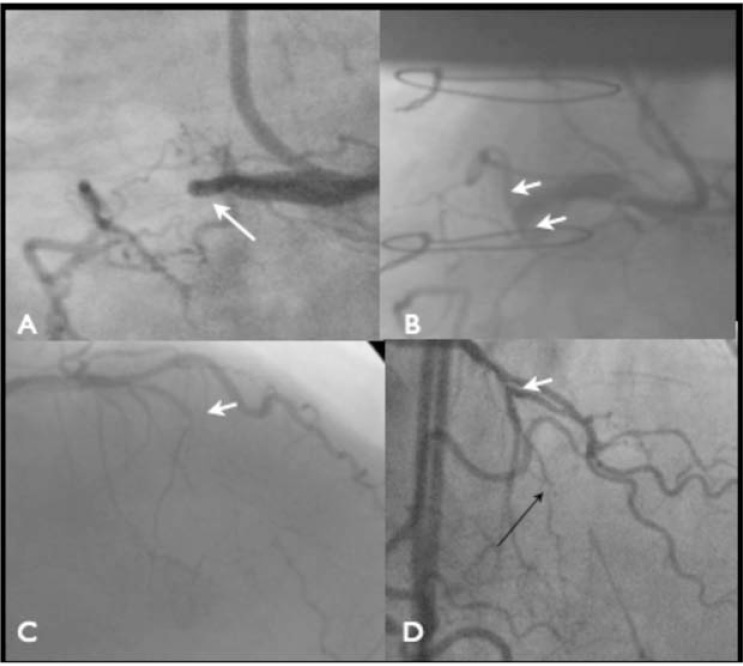
The proximal cap of the RCA (Panel A) and LAD (Panel C) occlusions are clearly visible. There is a septal branch near the occlusion (Panel C) but the cap is clearly visible. Compared to the ambiguity of similar RCA (Panel B) and LAD (Panel D) occlusions where the presence of multiple branches and the flush nature means it is hard to identify the exact origin of the occlusion. In Panel D the proximal cap is actually between the septal and diagonal (white arrow) the parallel septal branch is actually misleading (black arrow).

**Fig. (3):**
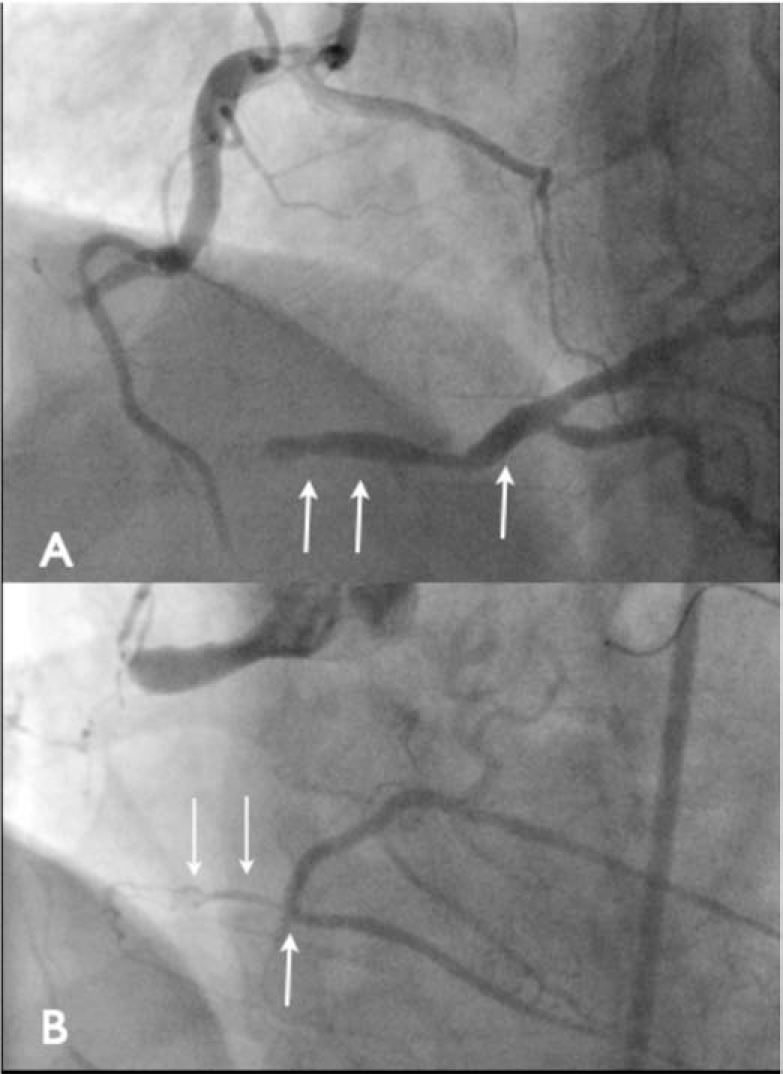
Two RCA occlusions, both have no proximal cap ambiguity and are >20 mm in length. The first image (Panel A) demonstrates a good landing zone with a large calibre distal vessel remote from side branches suitable for antegrade dissection re-entry. The second image (Panel B) demonstrates a poor distal landing zone with a heavily diseased vessel close to a major bifurcation. This is not an ideal landing zone for re-entry and a primary retrograde strategy would be recommended if there are interventional collaterals present.

1. Proximal cap: The presence of a clearly defined proximal cap (Fig. **2**), even if it is blunt rather than tapered, means it can be approached with confidence as the starting point and initial vessel course are clear. An ambiguous proximal cap refers to one where there are multiple branches, often collaterals, a flush occlusion and uncertainty as to the initial vessel course. The initial strategy is often influenced by this eg with a tapered proximal cap, especially with a short occlusion segment (<20 mm) an initial antegrade wire strategy is employed. Whereas with an ambiguous proximal cap and long occlusion segment a retrograde strategy is more likely to be the initial strategy. Finally, for cases where there is major ambiguity at the proximal cap, intravascular ultrasound (IVUS) should be used to help determine where the CTO segment begins [3].
2. Lesion length. When treating a CTO the lesion length is categorised as either<2 0 mm or ≥ 20 mm. Attempting CTO PCI on lesions greater than 20 mm was associated with significantly less likelihood of success using an antegrade wire escalation strategy with considerably longer procedure times [1]. These longer occlusions can more predictably be treated using a primary ADR or RDR strategy.
3. Distal cap and vessel characteristics. A relatively disease free, healthy vessel after a distal cap, clear of any major bifurcation would favour an ADR strategy (Fig. **3**). Whereas a small or heavily diseased vessel after the distal cap or if the distal cap is at the site of a major bifurcation would not. In this case a primary RDR strategy is preferable.
4. Interventional collaterals. This refers to the presence of collateral channels that can be accessed and crossed using a wire and microcatheter safely, that enter the distal vessel remote from the distal cap and facilitate retrograde wire escalation or RDR strategies. The absence of interventional collaterals precludes a primary retrograde strategy.

## ANTEGRADE STRATEGIES

If the CTO’s length is less than 20 mm then an antegrade wire escalation is the suggested initial strategy. In experienced hands a J-CTO easy lesion or score of 0 (and by default<2 0 mm) was associated with a high likelihood of successful crossing within 30 minutes (92.3%) [1]. Whereas with a lesion length of >20 mm and no other adverse features (J-CTO score of 1) the chances of crossing within 30 minutes was reduced to 58%. With additional adverse features and higher J-CTO scores this fell even further and a J-CTO score of >3 was extremely unlikely to involve successful antegrade wiring in under 30 minutes (22%). While antegrade wiring is feasible in these situations the procedures are often long and unpredictable even in the hands of experienced operators. 

When tackling a CTO of < 20 mm the set up should be the same as with a more challenging occlusion. This will allow smooth, rapid transition to an alternative approach if the initial strategy is unsuccessful. For example, using an 8F antegrade guide will allow rapid transition to an ADR strategy without having to change the access sheath and guiding catheter. The 8F guide catheter should sit well with with good coaxial alignment and passive support. In addition, using a guiding catheter for the contralateral injections rather than a diagnostic catheter will also facilitate smooth transition to a retrograde strategy. 

Other anatomical features such as a tapered versus blunt stump or the presence of branches or calcification will also affect ultimate success rates with this strategy. A tapered stump in the setting of a short occlusion length is a highly favourable anatomical feature. An ambiguous cap with multiple branches would suggest an increased likelihood of entering the occlusion sub-intimally. Subsequent re-entry back into the true lumen at the distal cap or beyond is unpredictable with a simple wire-based strategy. 

The initial wire should be ideally a tapered, polymer coated wire eg Fielder XT-A (Asahi Intecc, Nagoya, Japan) which has a tapered tip (0.010 inch) and is designed to traverse micro channels. Histological specimens demonstrate the presence of micro channels within the CTO segment [4] even if not radiographically visible. These are often 1 - 200 µm in diameter but may be as large as 500 µm. It is usually worth probing a short occlusion with a Fielder XT-A (tip 0.010 inch or 260 µm) as if a microchannel is located, this will significantly increase procedural efficiency. This attempt should not take long as it will become apparent very quickly whether the wire will progress or not. If the wire buckles over or fails to advance then a quick transition to an alternative wire will likely facilitate a more efficient procedure. If there is tortuosity within the CTO or the course is less well defined a Pilot 200 (Abbott Vascular, Santa Clara, CA) may cross the segment relatively quickly when a softer polymer jacketed wire has failed.

Histological data tell us that the proximal cap is often composed of calcified and resistant material. Subsequent wire choices should reflect this and operators should consider penetrative wire designed to puncture through the proximal cap eg Confianza Pro 12 (Ashai Intecc, Japan), Progress 200 (Abbott Vascular, Santa Clara, CA) or ProVia 15 (Medtronic Ltd) or other alternatives. Using a less penetrative wire may result in the wire buckling or deflecting towards the sub-intimal space. For these short CTO’s staying within the occluded lumen is preferable as it makes wiring of the distal vessel more likely.

These same principals apply to a CTO<20 mm which is approached from a retrograde direction. If the occlusion is short in length but with an ambiguous proximal cap but a clearly defined distal cap with good interventional collaterals and retrograde access is achieved then a retrograde wire escalation strategy can be employed. The wire choice and reasons for selecting them are the same as for antegrade wire escalation. 

### Antegrade Dissection and Re-entry Techniques

Antegrade dissection and re-entry occurs when a guidewire and microcatheter or specific device eg CrossBoss (Boston Scientific, Boston, USA) catheter are deliberately advanced within the sub-intimal space, past the CTO with a view to setting up for re-entry back into the true lumen of the distal vessel.

ADR was first reported by Colombo [5] who described the Subintimal Tracking and Reentry (or STAR) technique. With this technique the objective was to access the sub-intimal space proximal to the CTO, then to deliberately advance a knuckled guidewire along the sub-intimal space to the distal vessel, where reentry of all reasonably sized branches are attempted sequentially. This method was potentially unpredictable with a lack of control of the distal vascular bed being associated with unfavourable short and longer term outcomes. [6] The increased rates of late occlusion are mutlifactorial due to a combination of poor run off affecting flow within stents as well as the use of bare metal stents over long segments. These factors are the main reason this technique never became routine practice, but it was forerunner of contemporary and more standardized ADR techniques. 

This technique evolved into Limited Antegrade Subintimal Tracking (LAST) whereby after equipment had accessed the sub-intimal space and passed beyond the distal cap, a penetrative wire with a significant bend at the tip was used to immediately attempt to re-enter back into the lumen at this segment of vessel reconstitution. Again this technique was potentially unpredictable, mainly because once the sub-intimal space is accessed with large knuckle wires the degree of haematoma formation is uncontrollable. Haematoma formation and compression of the vessel lumen is undoubtably the achilles heel of antegrade dissection re-entry. Figure (**5**) shows an IVUS image from the subintimal space and demonstrates significant heamatoma in the space which has compressed the lumen. Re-entry using whatever method is much less predictable in this situation. The target (true lumen) has been compressed and the sub-intimal space is large so penetrating back into the distal vessel is difficult. 

**Fig. (5):**
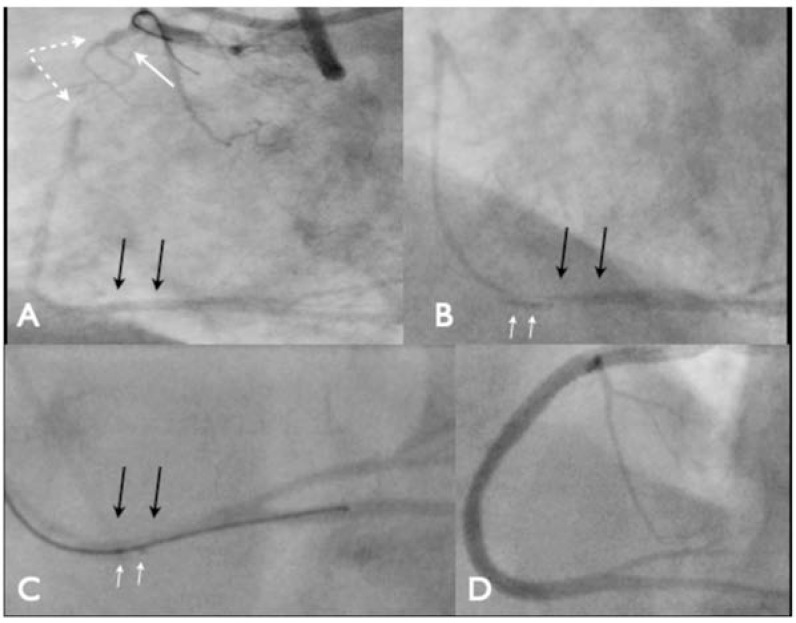
An occlusion of a RCA with ambiguity of the proximal cap (Panel A, white arrow), the occlusion is greater than 20 mm (dashed white lines) and there is a good landing zone remote from significant side branches (black arrows). After antegrade dissection the stingray balloon is positioned (Panel B) In this case the balloon lies below the vessel (white arrows). The Stingray wire exits the catheter just proximal to the most distal marker and re-enters the true lumen (Panel C). Final angiographic appearances (Panel D).

Scientific, Boston, USA) has been developed to facilitate ADR. With this device re-entry is more controlled and the procedure has become much more predictable [7]. The technology will be discussed in more detail in another companion article in this journal, but briefly the CrossBoss is a metal catheter and a 1 mm blunt head which moves in the subinitmal space ahead of a coronary guide wire. The blunt tip prevents the catheter from exiting the vessel architecture. Rapid rotation of the proximal torque device results in forward momentum through the extra-luminal portion of the vessel. The Stingray balloon is a 10 mm by 2.5 mm flat OTW balloon that is designed to deploy in the sub-intimal space and orientate around the vessel. There are two directional exit ports in addition to the end port. They allow exit either side of the balloon and are positioned just proximal to each of the two balloon markers. The Stingray wire (a 12g penetrative wire with a “barbed tip” to help catch the vessel) is directed through the port orientated towards the lumen thus facilitating re-entry. Once the correct orientation has been identified angiographically the Stingray wire is advanced through the appropriate port into the lumen. The lumen can be wired with this wire “stick and drive” or more commonly a more steerable wire is used in a “stick and swap” strategy (Fig. **5**). 

## ANATOMICAL CONSIDERATIONS AFFECTING ANTEGRADE DISSECTION RE-ENTRY STRATEGIES

### Distal Landing Zone

The main anatomical features affecting a decision to consider an ADR strategy are the quality and calibre of the distal landing zone and its proximity to major side branches.

If the distal landing zone of the target vessel is of good calibre and size and remote from significant side branches/bifurcations then an antegrade dissection re-entry strategy with a CrossBoss and Stingray would be appropriate (Fig. **3**, Panel A).

However if the distal vessel is heavily diseased, small in calibre or at the point of major side branches then antegrade dissection re-entry is not ideal (Fig. **3**, Panel B). In this situation a primary retrograde approach is indicated, provided there are interventional collaterals present. 

### The Proximal Cap 

**Fig. (6):**
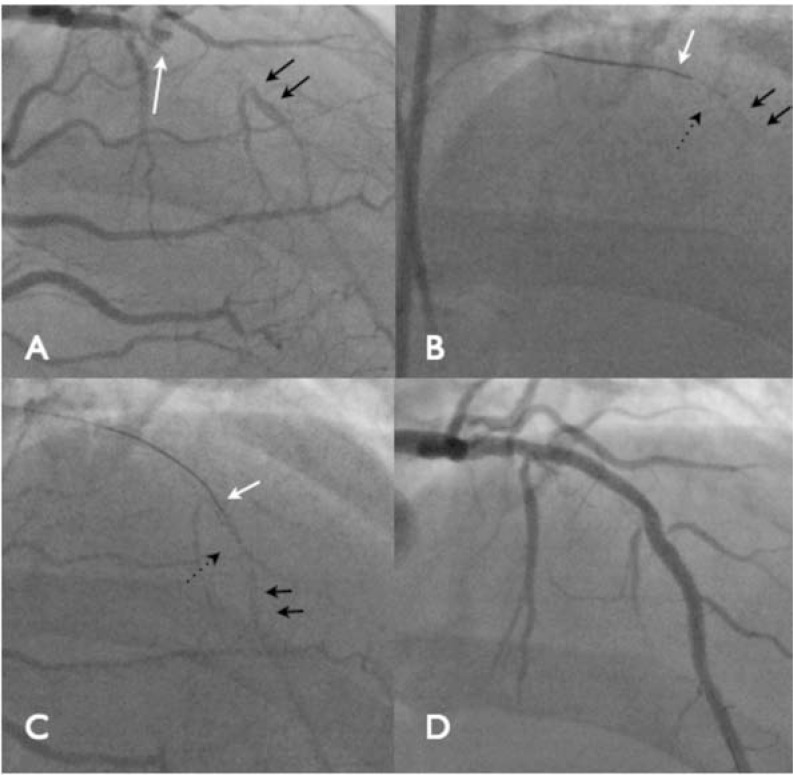
There is an LAD occlusion, the proximal cap is unambiguous (Panel A, white arrow), the lesion is greater than 20 mm, the distal landing zone is good (black arrows) and interventional collaterals are present from the RCA. The occlusion is tackled with a CrossBoss catheter directly (Panel B, black dotted arrow). A workhorse wire is used to access the LAD then withdrawn into the catheter as it engages the occlusion (white arrow). The CrossBoss advances within the occlusion (Panel B) towards the distal landing zone (black arrows). It passes from true lumen to true lumen (Panel C) with an angiogram demonstrating that the tip is luminal. Final angiographic appearance (Panel D).

A proximal cap that is clearly identified favours an initial ADR approach - especially if the distal landing zone is of good calibre. When there is no ambiguity at the proximal cap the lesion may be approached directly with the CrossBoss catheter (Fig. **6**), although calcification and tortuosity in the proximal vessel may limit this approach. As compared to a knuckled guide wire the CrossBoss catheter design will limit the size of the dissection plane and subsequent haematoma formation. Then when the Stingray balloon is then deployed it is more likely to encompass the lumen, thus increasing the chances of success with wire re-entry.

**Fig. (7):**
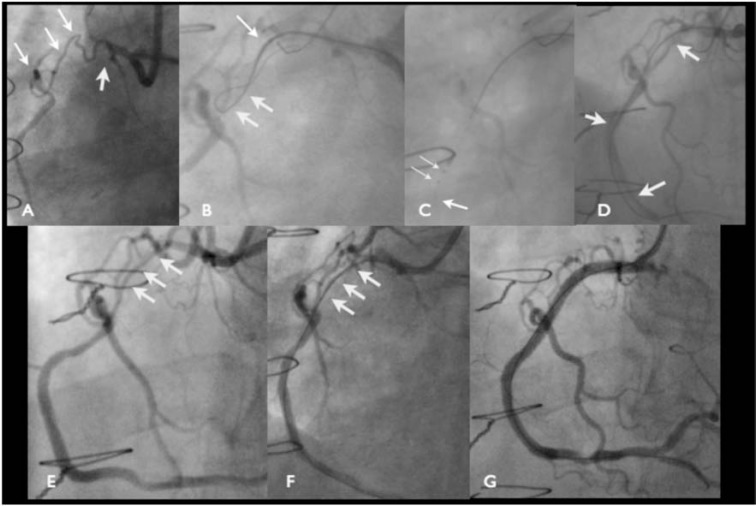
An occluded RCA with an ambiguous proximal cap (Panel A, white arrow), occlusion length >20 mm, good landing zone and a noninterventional collateral (white arrows). To overcome the ambiguity at the proximal cap (Panel B, single white arrow) a penetrative wire is used to puncture the cap, the corsair is advanced into the occlusion and a Fielder XT knuckle wire is used to define the anatomy. However the knuckle that forms is very large (white arrows) and haematoma develops. By the time the CrossBoss is used and then Stingray balloon deployed (Panel C, white arrows) the distal vessel is compressed and very faint (single white arrow). Re-entry is very difficult in this situation and ultimately unsuccessful, there was extensive dissection in the vessel on final angiographic shot. After 8 weeks the patient was re-admitted for another attempt. In fact the vessel had re-cannalised (Panel E, white arrows) suggesting that during the index procedure re-entry had been successful but not appreciated due to the extensive haematoma. The RCA was easily wired (Panel F) and stented with excellent final result (Panel G).

Alternatively, if there is significant ambiguity of the proximal cap a primary retrograde approach may be favoured as the initial strategy. ADR can still be attempted but care must be taken when crossing the proximal cap and proximal vessel as the architecture may be completely undefined and the risks of a complication are much higher under these circumstances. After puncturing an ambiguous cap with a penetrative wire, we would recommend that a Corsair or alternative microcatheter is advanced a very short distance into the occlusion segment and then a knuckle wire is introduced and advanced. This manoeuvre is much more likely to keep equipment safely within the vessel architecture. The CrossBoss catheter (Boston Scientific, Boston, USA) can be used to traverse the last few cm’s before the target landing zone to control the size of the dissection plane in this segment. This process can be unpredictable for several reasons: controlling the knuckle size can be difficult, especially is a lot of force is required to initiate the knuckle. This can lead to more extensive haematoma formation. This is also true if balloon dilatation of the cap and proximal vessel are required to aid CrossBoss access to the sub-intimal space. Extensive haematoma formation will compress the distal lumen making re-entry more difficult (Fig. **7**). 

### Occlusive Restenosis

**Fig. (8):**
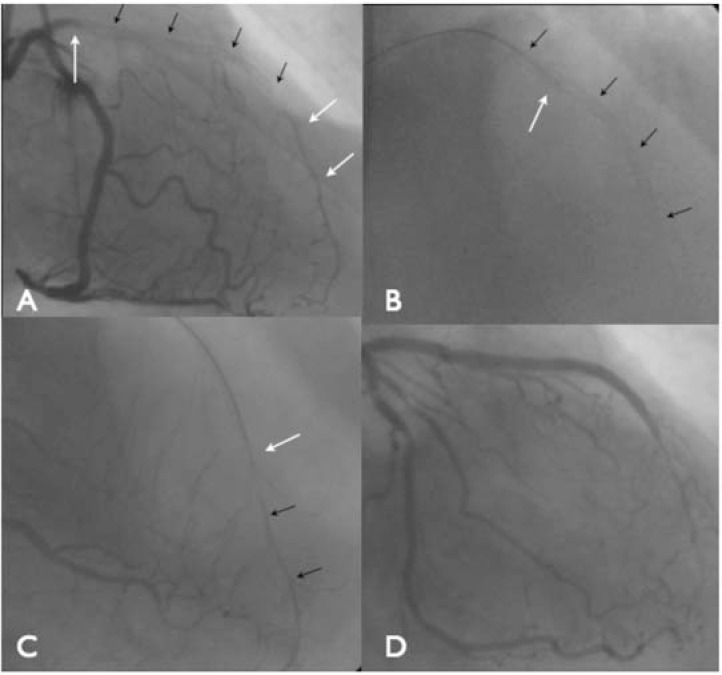
There is a long area of occlusive stents within the LAD (Panel A, Black arrows). The occlusion extends back to the origin of the stents (White arrow). The distal vessel fills from interventional septal collaterals from the RCA (White arrows). The occlusion is traversed with a CrossBoss catheter (Panel B, white arrow) safely and efficiently, this is visible within the stents (black arrows). The CrossBoss (Panel C, white arrow) enters the true lumen distally directly (black arrows). An acceptable final result is achieved efficiently (Panel D).

Occlusive restenosis represents 5-10 % of all CTO’s [8] but with previously poor re-cannulation rates [9]. They do appear to be a particular sub group of chronic occlusion which is particularly pre-disposed to the use of CrossBoss catheter (Boston Scientific, Boston, USA) (Fig. **8**) and we have reported >80% success rates with low complication and short crossing times in occlusive restenosis using this device [10].

### Retrograde Strategies 

Retrograde approaches to CTO PCI can frequently be more complex and prolonged than either simple or more complex contemporary antegrade approaches. Nevertheless, the retrograde approach is a fundamental and necessary skillset that must be learnt for a successful CTO PCI programme. Operators will need to pay specific attention to a number of additional features during these cases that include careful monitoring of anticoagulation, appropriate guide catheter selection (both in terms of diameter and the support offered) as well as familiarity with a range of micro-catheters and their manipulation (particularly the Corsair microcatheter). The specific technical approaches and challenges related to retrograde CTO PCI are covered in more detail in a companion article in this issue of the journal. 

The key to a successful retrograde PCI procedure is the collateral channel from the donor vessel. A classification system for collateral channels has been proposed based on a modified Rentrop system: CC grade 0: no continuous connection, CC1: thread-like continuous connection, CC2: side branch-like connection [11]. Whilst these features may help predict collateral flow reserve, the presence of CC0 or CC1 collaterals does not mean the non-angiographically visible or very tiny channels should not or indeed cannot be used to obtain successful retrograde access. Therefore, the practical applicability of this classification is limited in terms of approaching retrograde CTO PCI. 

There are 3 types of retrograde channel that may be of use. These include grafts (internal mammary arteries should be approached with due care and handled extremely carefully as a complication can have catastrophic implications). Venous grafts often represent a good option for retrograde access and diseased, but patent grafts often offer an excellent opportunity to allow operators to learn how to perform retrograde CTO PCI procedures. Occluded vein grafts are also 

often crossable, [12] therefore, these conduits also deserve careful assessment during diagnostic angiography. Septal collaterals usually offer safe access to the distal portion of the occluded vessel. However, these vessels may have marked anatomical challenges, as described below, making them potentially more difficult and less reliable to cross than may be assumed from the initial angiographic evaluation. On the positive side, the consequences of limited and small perforations are typically minimal, limited dissections usually have no clinical sequelae and these channels are amenable to dilation with small calibre balloons if secondary equipment cannot be advanced to the occluded vessel. Epicardial collaterals occur in a wide range of states that vary from very large calibre vessels to tiny, threadlike vessels. The more challenging epicardial collaterals should also be approached with caution, particularly if the CTO PCI operator is early in the retrograde learning curve. The major risk of wiring/crossing epicardial collaterals is of perforation. This can have potentially deleterious implications, especially in a patient who has not had previous cardiac surgery, where the pericardium is intact. Early cardiac tamponade is a potential complication under these circumstances. Finally, in large calibre epicardial collaterals, especially those with significant tortuosity, the operator should be aware that passing a guidewire and/or microcatheter may precipitate significant ishcaemia during the case and that this may cause distress and discomfort for the patient during the procedure. This is in contrast to crossing septal channels, where there are usually multiple connections and this issue is much less common. 

### Retrograde Access via a Saphenous Vein Graft 

Patent venous grafts offer the benefits of being easily accessible, allowing visualization of the target vessel and they also usually offer a simple conduit to pass a guidewire to the true lumen of the distal segment of the occluded native vessel. Occluded vein grafts are also potentially of use, particularly recently occluded vessels (although in the experience of the authors vein grafts that have been occluded for 12-24 months may still offer a retrograde option). Under the latter circumstances, our experience is that a hydrophillic jacketed guidewire such as a Pilot 200 (Abbott Vascular) in combination with a Corsair microcatheter will usually offer the safest method of crossing the graft. Ideally, the operator should aim to find another donor vessel that fills the distal segment of the target vessel during access via an occluded graft. This allows an angiographic assessment of when the target vessel has been reached with a guidewire/microcatheter as well as where to advance the wire retrogradely into the occluded target native vessel. This will usually mean switching this latter catheter out for a guide that is then seated in the target vessel to allow antegrade access. Finally, when the wire and microcatheter have been advanced to beyond the occluded segment of the target vessel, the operator should anticipate significant angulation at the vein graft anastamosis and have strategies or equipment prepared to overcome this obstacle. 

### Retrograde Access via Septal Collaterals

**Fig. (9):**
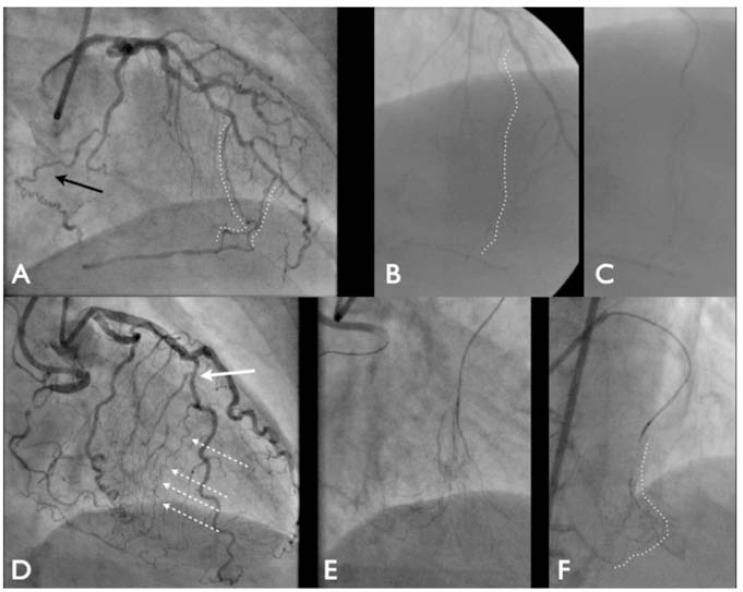
An example of septal connections. Panel A: Markedly tortuous epicardial non-interventional collateral in the AV groove (black arrow) with a moderately tortuous septal (left dotted white line) and a very favourable straight septal connection from the distal LAD (right dotted white line). Panel B: A straight and single connection from the mid LAD to the distal PDA. Panel C: Corresponding tip injection with the Corsair placed selectively in the same vessel demonstrating multiple small branches from this vessel. Panel D: A tortuous LAD with multiple connections to the RCA. The solid arrow demonstrates a channel with a retroflex origin arising just beyond a significant curve in the main vessel that is very difficult to access. The next distal septal vessel has marked tortuosity through its course. Panel E: Selective tip injection in a proximal septal vessel demonstrating a multitude of different branches within the vessel. Panel F: A different angiographic projection of the same septal in panel E. Marked angulation can be appreciated at the transition from the LAD to RCA territory, with a sharp 90° bend noted (dotted white line).

A series of anatomical assessments are necessary before selecting a septal collateral for attempted retrograde crossing. Firstly, the donor vessel should be assessed. Excessive tortuosity should prompt the thought that the main vessel could be kinked or straightened during passage of the Corsair. The presence of severe lesions in the main artery may precipitate ischaemia during the procedure and may require PCI before attempting retrograde access for the occluded vessel. The angle that the septal branch arises at is also an important feature. The combination of excessive early tortuosity combined with marked angulation at the septal origin (especially if the vessel arises within or just after a very tortuous segment from the main vessel) may make the collateral non-useable. Tortuosity within the septal branch itself may also lead to significant difficulty in crossing the collateral. There are frequently a number of branches within the septal channel that may not always be obvious from standard angiography. In addition, marked angulation at the transition from LAD to RCA territories (from either direction) can also make wiring, but more so Corsair advancement extremely difficult. This latter issue can usually be overcome by dilation of the septal channel with a small calibre semi-compliant balloon. Finally, the angle of connection back into the recipient vessel can also lead to challenges. The coronary anatomy of the recipient vessel is also pertinent. A number of “generic” challenges can arise under these circumstances (see below, Fig. **9**). When a septal collateral connects with the occluded vessel very close to the distal cap, especially if there is a bifurcation in this area, then engaging the occluded segment can be challenging. 

Once the most favourable septal collateral has been chosen, the septal is typically wired with any workhorse guidewire. Once the microcatheter has been advanced into the proximal channel then the workhorse wire is removed and swapped out for a guidewire that is specifically shaped for septal collateral crossing. There are then 2 broad approaches to crossing septal collaterals. One is to “surf” the channels by gently advancing the wire forwards. As long as the wire makes progress and meets no resistance the channel can be safely explored to see if it advances into the target vessel. Excessive whipping or motion of the distal wire tip usually signifies entry into a ventricular cavity. Withdrawal of the guidewire under these circumstances almost universally occurs without adverse consequences. It should be recognized that there are arterial-arterial, arterial-venous and arterial-chamber connections and that there is an element of conditional probability that dictates successful passage of the “surfing” guidewire through septal collaterals. Therefore, different channels should be deliberately assessed and different bends on the wire tip may be needed to facilitate access to a number of channels. Another approach to septal crossing is to make a much more specific assessment of the anatomy. This can be safely achieved by “tip injections” of contrast through the micro-catheter to delineate the septal channel that is being interrogated. When this is being performed, then the septal channel may need to be assessed in different angiographic views to completely understand tortuosity and branching. It is useful to inject contrast using a 2 or 3ml luer-lock syringe after aspirating the catheter to ensure that wire withdrawal has not entrained any air. Flushing the micro-catheter separately after the contrast injection with 2-3mls of heparinized saline will expel the remaining contrast from its lumen. This will allow a second image to be obtained in another view, whilst also clearing the lumen and preventing it from becoming resistant to wire manipulation and passage due to the viscosity and stickiness of the contrast media. When a detailed anatomical assessment of a specific channel has been made, then wires are usually shaped to adapt to the requirements of the target conduit and they should be steered carefully and intentionally into these channels.

**Fig. (10):**
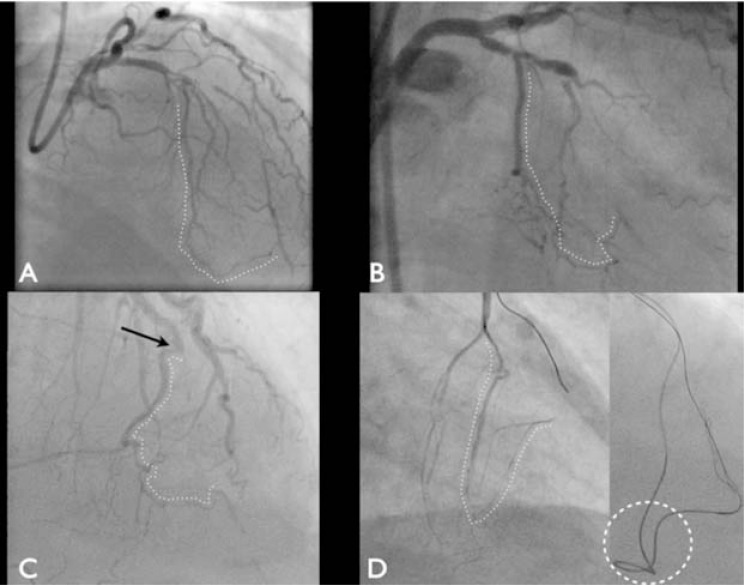
Examples of septal loops. Panel A: An obvious and straight connection from the proximal LAD to the distal true lumen. Panel B: A moderately tortuous connection. Panel C: A septal loop with a diseased and angulated entry and marked tortuosity. Panel D: Corresponding tip injection in a different angiographic projection demonstrating marked angulation at the transition back towards the distal vessel (dotted white line showing V like shape). After passing the Corsair catheter around the septal loop, the microcatheter develops a figure of 8 configuration and manipulation is usually very difficult under these circumstances. Substantial care needs to be taken not to damage the septal channel during advancement of the Corsair.

The issue of septal loops also deserves some consideration (Fig. **10**). When present, a proximal septal connects to a more distal septal branch of the LAD and may allow access to the true lumen beyond the occlusion. In our experience, there can also be significant tortuosity within these segments. We have found it safest to only advance Corsair catheters through these loops, as the construction of this microcatheter prevents kinking and affords protection to the collateral channel itself. Rarely, it can be very difficult to advance a the Corsair catheter through a septal loop back towards the proximal vessel. If the CTO segment has been successfully crossed but wire externalization is not feasible, “tipping in” (advancing a Sion guidewire antegradely into the retrogradely sited Corsair lumen) can facilitate wire passage to the true lumen of the distal vessel. In general, the presence of an externalized guidewire provides a very secure rail to facilitate completion of the PCI and this is our preferred option for completion of the vast majority of cases. 

Major complications related to septal collaterals are relatively uncommon [13] although significant haematoma, [14] tamponade [15] and ventricular septal defects have been reported in rare cases. [16] Our experience is that marked angulation and tortuosity witihin the septum are major predisposing factors to potentially adverse outcomes and that if there is major resistance to advancing equipment, then alternative channels should be sought. 

### Retrograde Access via Epicardial Collaterals

Epicardial collaterals can also range from being very straight connections with limited branching, to demonstrating high-frequency tortuosity with a “telephone cord” like appearance. Whilst wiring large calibre and straight epicardial collaterals is usually straightforward, small calibre tortuous vessels with extreme angulation at either the entry-point or exit can offer the most extreme challenge. One suggested rule of thumb for tortuous epicardial collaterals is that those with a ratio of the collateral loop width divided by the distance between two consecutive collateral loops of >2, may be extremely challenging or impossible to wire [17]. Brilakis & gang CCI 2012]. The more high frequency loops there are, the more extreme the challenge becomes. 

**Fig. (11):**
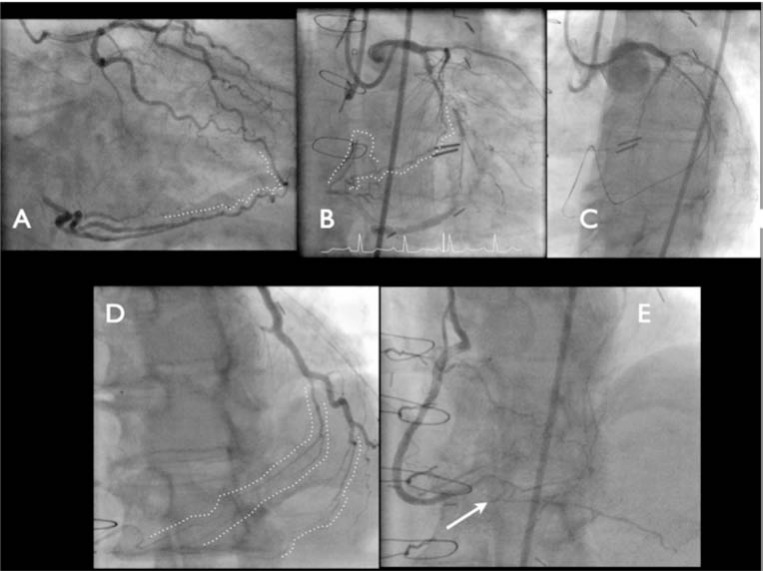
Examples of interventional epicardial collaterals. Panel A: An apical connection from a LAD to the RCA. This vessel is of a good size (CC2) and has only mild tortuousity. Similar channels can be a cause of ischaemia during the case and operators should be prepared for this. Panel B: There are 2 connections from the LCx through the AV groove to the distal RCA. Whilst there is significant tortuosity and angulation. However, these channels can be crossed with due care (Panel C). Panel D: Smaller straight but threadlike CC1 collaterals from a grafted LCx with multiple connections to the occluded RCA. Panel E: The same vessel after reconstruction. The distal RCA is tented at the site of the old graft anastamosis (arrow) and it is frequently easier to pass wires and retrograde equipment into the occluded graft, rather than the target vessel.

Ipsilateral epicardial collaterals also deserve consideration. These vessels are frequently of use and time should be taken during diagnostic angiography to ensure that all potential options are explored. For example, care should be taken to ensure that the proximal conus branch of the RCA is also imaged as this may offer a connection to the distal RCA. Similarly, existing left to left epicardial channels can offer equally good connections to the distal true lumen. A range of epicardial collaterals with varying degrees of difficulty are presented in (Fig. **11**).

Perforation of an epicardial collateral can represent a challenging clinical scenario. In such cases, the epicardial collateral may fill not only from the donor vessel but also from other collaterals and via the occluded target vessel as well. Operators should be familiar with a variety of treatment options including covered stents, coil embolization, injection of autologous fat [15] and should also liaise closely with surgical colleagues in case operative repair is required. 

### Recipient Vessel Issues

**Fig. (12):**
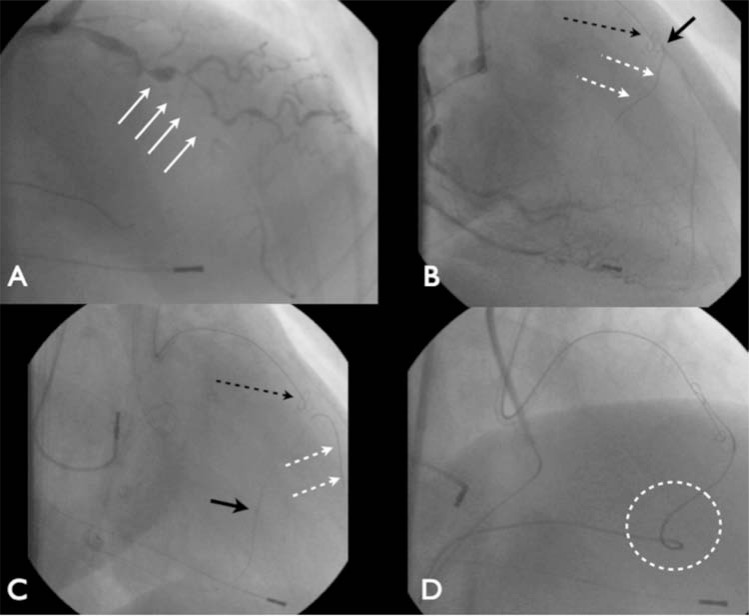
Potential difficulties with recipient vessels. Panel A: LAD occlusion in the mid vessel. There is considerable proximal cap ambiguity with a 25mm occlusion. Panel B: Antegrade knuckle wire in the LAD architecture (black arrow). A wire has been passed retrogradely (white arrows) through a non-visible CCO septal connection but joins the LAD right at the distal cap of the CTO. Panel C: The septal channel requires balloon dilation (solid black arrow) and the retrograde wire passes preferentially into the patent distal LAD (white arrows). Panel D: Overlapping knuckle wires are present to facilitate a reverse CART. There is significant tortuosity and angulation at the transition from the RCA to LAD territories leading to the Corsair configuration as shown (circled).

Severe calcification at the distal cap can make penetration of the CTO segment very difficult and enhancing support of the microcatheter is challenging under these circumstances. The presence of grafts (where the graft itself has not been used to gain retrograde access) on the recipient vessel can lead to significant tenting or distortion and make retrograde wire passage to the graft itself more likely than to the target vessel itself. Severe calcification and tortuosity within the occluded segment can make passage of retrograde equipment challenging. It is also crucial to recognize when the equipment that is passing retrogradely is passing out of the architecture of the main vessel structure and tracking towards side branches. Inadvertently following side-branches with guidewires and secondary equipment will increase the risk of coronary perforation during the case (Fig. **12**).

## CONCLUSIONS

In contemporary practice there are 3 main methods for opening a CTO, wire escalation from an antegrade or retrograde direction, antegrade dissection re-entry and retrograde dissection re-entry. Published data indicates that success rates range between 60 - 75% for antegrade wire escalation only strategies [18] and that even in expert hands collateral channel crossing was successful only 84% of the time [19]. Having the full range of CTO interventional skills is required to maximize the chances of success for patients.

While the hybrid algorithm was developed by the US operators it has gained popularity in Europe and Japan. Recently Tsuchikane [19] refers to the Hybrid algorithm as a consistent framework for physician education and teaching and a template upon which future guidelines for CTO PCI practice will be based.

When deciding which strategy to employ initially the anatomical features described above will best advise the method with the greatest likelihood for procedural success. Hopefully this will result in improved procedural safety and efficiency. Careful and detailed assessment of all aspects of the coronary anatomy are key to directing the initial procedural approach, but will also inform the operator regarding secondary and tertiary interventional approaches to result in a successful outcome for the patient. Understanding each feature is key and assessment is crucial to success. 

## Figures and Tables

**Fig. (4):**
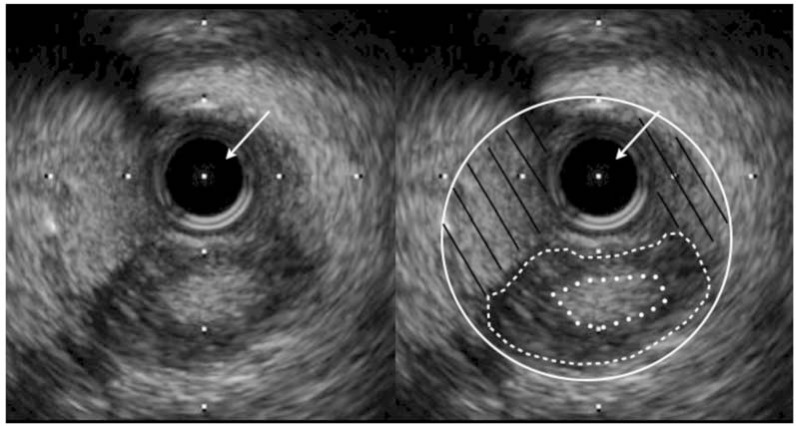
An IVUS image of a haematoma in the dissection plane causing lumen compression. The IVUS catheter (white arrows) is visible. The adventitia (white line) and the dissection plane with haematoma within the subintimal space vessel (black lines) are demonstrated. The vessel media (white dashed lines) and the compressed true lumen (white dots) are visible.
